# Physiotherapeutic scoliosis-specific exercises performed immediately after spinal manipulative therapy for the treatment of mild adolescent idiopathic scoliosis: study protocol for a randomized controlled pilot trial

**DOI:** 10.1186/s13063-020-05000-y

**Published:** 2021-01-14

**Authors:** Li Wang, Chun Wang, Ahmed S. A. Youssef, Jiang Xu, Xiaolin Huang, Nan Xia

**Affiliations:** 1 Department of Rehabilitation Medicine, Tongji Hospital, Tongji Medical College, Huazhong University of Science and Technology, Wuhan, China; 2 Department of Rehabilitation Medicine, Shantou Central Hospital, Shantou, China; 3 Basic Science Department, Faculty of Physical Therapy, Beni-Suef University, Beni-Suef, Egypt

**Keywords:** Spinal manipulative therapy, Physical exercise, Adolescent idiopathic scoliosis, Somatosensory evoked potentials

## Abstract

**Background:**

Spinal manipulative therapy is commonly used in the treatment of adolescent idiopathic scoliosis. Some therapists also rely on physiotherapeutic scoliosis-specific exercise (PSSE). Combining these two modalities seems reasonable, but the effectiveness of this combination has never been rigorously tested. Here, a protocol for a pilot study is proposed to determine the feasibility of conducting a larger randomized trial. The pilot study was designed to test the hypothesis that spinal manipulative therapy followed by PSSE is more effective than PSSE alone in improving the Cobb angle, sensorimotor integration, the angle of trunk rotation (ATR), body symmetry, and quality of life.

**Methods:**

The protocol describes a randomized controlled pilot trial with 40 subjects divided into study and control groups. Both groups will receive 8 weeks of PSSE, but the study group will also receive spinal manipulative therapy during the first 2 weeks before PSSE. The primary outcome will be an estimate of the feasibility of conducting a full-scale experiment. The influencing factors will be the time to complete enrollment, the recruitment rate, subject retention, and adherence to the treatment allocations. The secondary outcomes that will be used to assess the efficacy of treatment will include the Cobb angle, somatosensory evoked potentials, ATR, three-dimensional postural parameters, and scores on the 22-item Scoliosis Research Society outcomes questionnaire. The Cobb angle will be measured at baseline and at the end of 8 weeks of training. The somatosensory evoked potentials will be measured at baseline and at the end of 2 weeks of training. The ATR, three-dimensional postural parameters, and scores on the 22-item Scoliosis Research Society outcomes questionnaire will be measured at baseline and at 2 weeks, 4 weeks, and 8 weeks of treatment.

**Discussion:**

This study will inform the design of a future full-scale trial. The outcomes will provide preliminary data about the efficacy of the combination of spinal manipulative therapy and exercise in treating scoliosis.

**Trial registration:**

Prospectively registered at Chinese clinical trial registry, ChiCTR1900027037. Registered on 29 October 2019.

http://www.chictr.org.cn/edit.aspx?pid=44954&htm=4

**Supplementary Information:**

The online version contains supplementary material available at 10.1186/s13063-020-05000-y.

## Background

Adolescent idiopathic scoliosis (AIS) is characterized by structural, lateral, and rotated curvature(s) of the spine in individuals aged 10–18 years old, and AIS accounts for 70–80% of the scoliosis cases [1, 2]. The reported incidence of AIS in China is approximately 2.4–5.1% [3, 4]. Its etiology remains unclear [5, 6]. Importantly, AIS is very progressive. As the degree of scoliosis increases, it may cause back pain, psychological problems, cardiopulmonary dysfunction, and other issues, severely affecting the physical and mental health of teenagers [7–10]. The prevalence of back pain has been reported to range from 22.5 to 68% [7, 11]. Recent studies have found that AIS patients are more prone to not only musculoskeletal problems but also problems of balance, sensorimotor control, sensorimotor integration, and motor control than are normal subjects [12–15].

Many nonsurgical treatments have been proposed to delay the need for surgery, with the goal of preventing or reversing the progression of the spinal curvature, but few of these treatments have been included in the guidelines recently published by the International Scientific Society of Scoliosis Orthopedic and Rehabilitation Treatment (SOSORT) [1]. Braces and physiotherapeutic scoliosis-specific exercises (PSSE) are the only two treatments supported by level I evidence and accepted by the SOSORT. Due to the physical discomfort of wearing a brace and the psychological distress resulting from its appearance [16–18], braces are recommended only for growing patients with progressive idiopathic scoliosis exceeding 25°. PSSE, however, is recommended as the first choice for mild AIS (Cobb angle < 25°) to halt the progression of the deformity [1]. Simultaneous, active, three-dimensional PSSE for correction is emphasized to improve sensorimotor integration and motor control [19].

Spinal manipulative therapy (SMT) is commonly used to treat musculoskeletal dysfunction. It has a significant curative effect on pain, flexibility, and mobility [20–22]. However, it has not yet been shown to be effective in treating AIS. Contradictory research results have been reported, and the research has often been of poor quality. Reports of SMT reducing the Cobb angle and trunk rotation have often been included in case reports or pre-and-post studies [23, 24]. One randomized controlled trial (RCT) showed that manual therapy did not affect trunk morphology or spine flexibility. However, in that RCT, the patients received manual therapy without PSSE [25]. Another pilot RCT confirmed the feasibility of performing RCTs on manipulation for scoliosis and compared chiropractic care with standard medical care [26]. There is a great need for high-quality RCTs to verify the efficacy of manual therapy in treating AIS.

Interestingly, there is evidence that high-velocity, low-amplitude (HVLA) spinal manipulation can induce cortical plasticity which improves sensorimotor integration and alters motor control [27]. Changes in motor evoked potentials [28] and sensory evoked potentials [29] as well as changes in the amplitude of maximum voluntary contractions [30] have been observed after HVLA manipulation. Together, these observations together suggest that HVLA manipulation alters somatosensory processing [31] and improves motor performance for at least 60 min after a typical HVLA manipulation session [32]. Thus, we hypothesize that active PSSE during the period of improved sensory-motor integration after SMT might thereby improve the training effect and lead to better a posture and quality of life.

A randomized and controlled pilot trial was therefore designed to test this idea. The pilot trial will investigate the feasibility of conducting RCTs on this topic with regard to recruitment (e.g., time to complete enrollment, recruitment rate), subject compliance with the study protocols, and adverse events. A secondary aim is to investigate the effect size of SMT combined with PSSE by comparing a group receiving SMT with PSSE and a group receiving PSSE only. The outcomes are the Cobb angle, somatosensory evoked potentials (SEP), the angle of trunk rotation (ATR), three-dimensional postural parameters (3-DPPs), and the 22-item Scoliosis Research Society outcomes questionnaire (SRS-22) score.

## Methods/design

### Study design

**Fig. 1:**
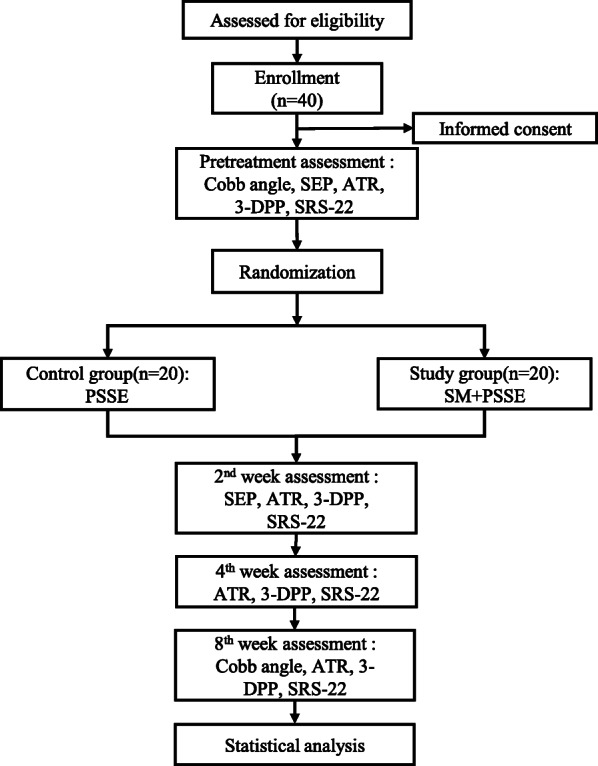
The flow diagram for this trial

The study will be a nonblinded pilot RCT with two parallel groups. The protocol will follow the Declaration of Helsinki and will be conducted according to the standard protocol items: recommendations for interventional trials (SPIRIT) guidelines and the World Health Organization trial registration data set (version 1.2.1) guidelines for clinical trial protocols (see Additional file 1 and Additional file 2). It will be conducted in the Rehabilitation Department of Tongji Hospital at Huazhong University of Science and Technology in Wuhan, China. AIS subjects will be recruited from the hospital’s outpatient rehabilitation department. To recruit more subjects, we will post study-related advertisements in the Outpatient department and WeChat app. A flow diagram is shown in Fig. 1.

### Eligibility

The inclusion criteria will be as follows [33]:

1. Age of 10–18 years.
2. A diagnosis of idiopathic scoliosis.
3. Cobb angle of 10–25°.
4. Risser sign of 0–2.
5. Agree not to receive any other treatments for scoliosis such as brace and insoles during the study period.

The exclusion criteria will be as follows:

1. Contraindications for exercise, such as cardiovascular or respiratory insufficiency.
2. Contraindications for SMT, including inflammation, infection, advanced degeneration, congenital malformations, trauma, and cerebrovascular anomalies [34].
3. A history of spinal surgery.
4. A history of scoliosis treatment involving surgery, a brace, exercise, etc.

In addition to the simple unwillingness to continue, factors that can prevent a subject from continuing to participate, such as a new diagnosis of a severe disease, will be considered valid grounds for withdrawal.

### Randomization and blinding

The physician will explain the whole content of the trial to the candidates who meet the eligibility criteria in a separate room. Written informed consent from both subject and their statutory guardians are required. Their demographic information and medical history will then be recorded.

Each subject will be assigned to either the study or the control group using simple randomization with a 1:1 allocation. The random sequence will be generated by an investigator using an electronic random sequence generator (www.random.org). The numbers in the random sequence will be sealed in consecutively numbered, opaque envelopes. When required, the envelopes will be opened by a person who will not be involved in the intervention, assessment, or statistical analysis.

**Fig. 2:**
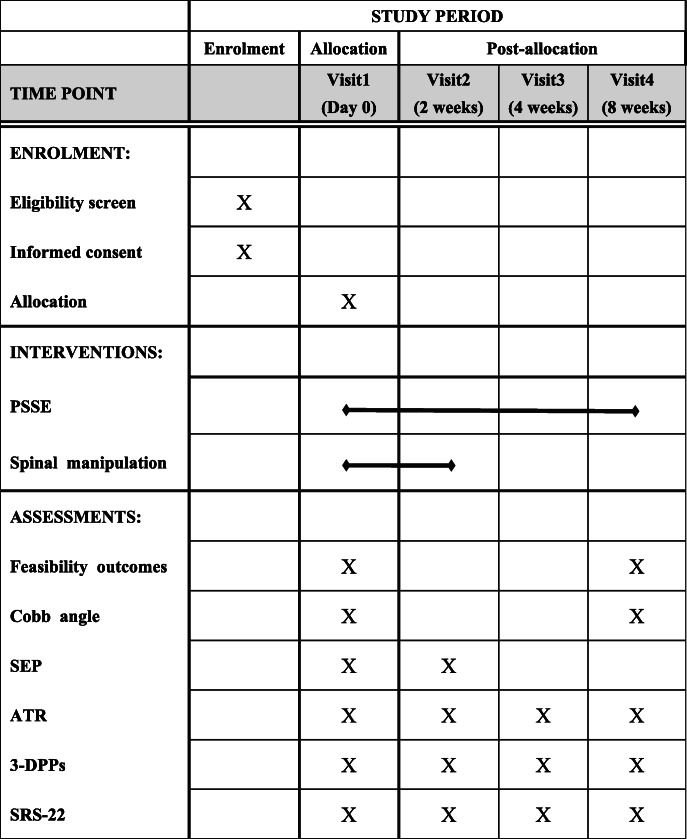
Schedule of enrolment, interventions, and assessments throughout the trial. PSSE, physiotherapeutic scoliosis-specific exercise; SEP, somatosensory evoked potentials; ATR, angle of trunk rotation; 3-DPPs, three-dimensional postural parameters; SRS-22, the 22–item Scoliosis Research Society Outcomes Questionnaire

Due to the nature of the interventions, blinding of the therapists and subjects to the intervention type will be impossible, so only the assessors and those performing the statistical analysis be blinded to the allocation results. To ensure that the group allocation results remain concealed as much as possible, the assessors will process the assessments in a separate room, and the statistical analysis will not include the information about the subjects and their allocations. The schedule for the treatments and outcome assessments is presented in Fig. 2.

### Intervention

#### Physiotherapeutic scoliosis-specific exercises

**Fig. 3:**
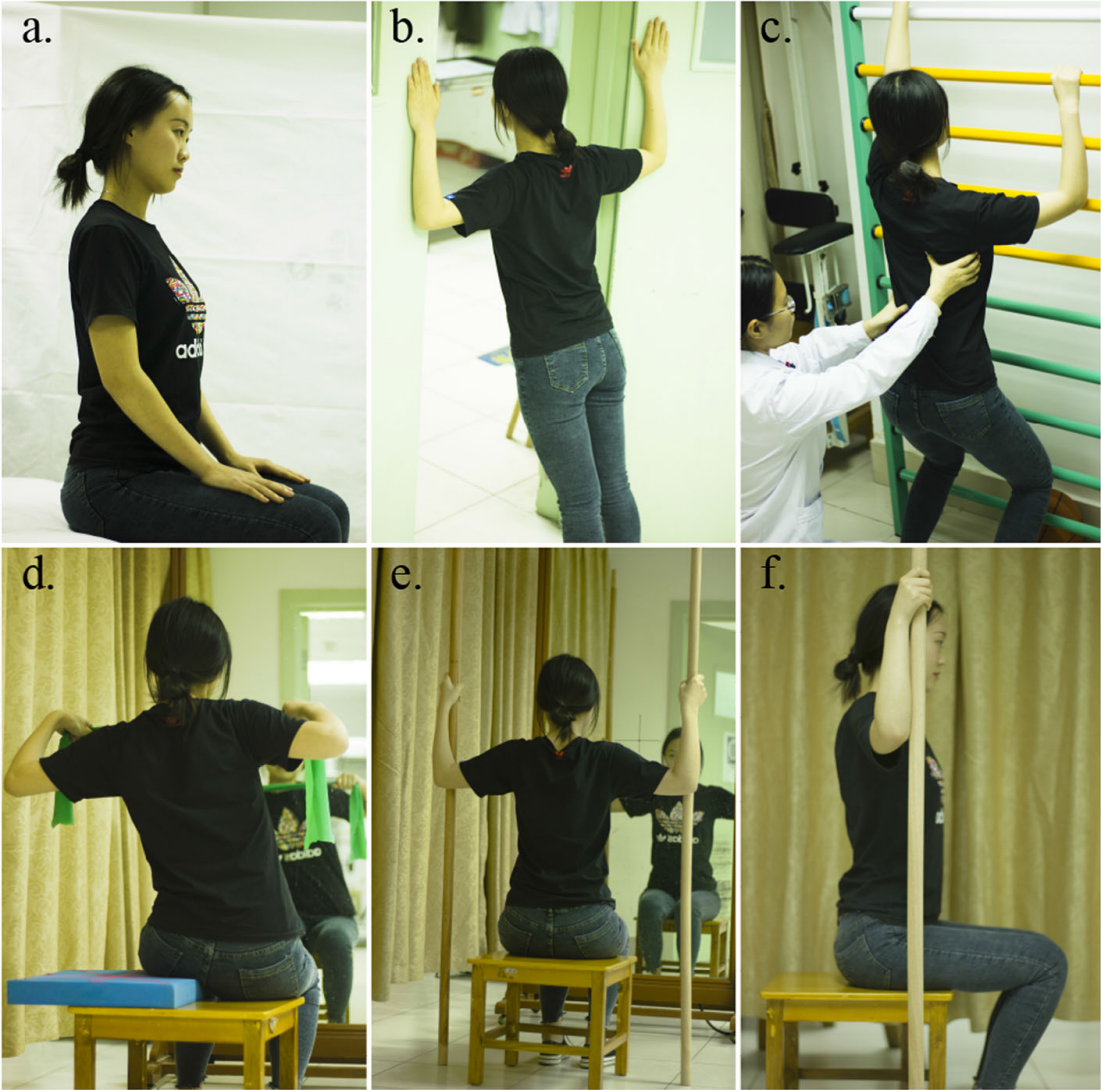
Physiotherapeutic scoliosis-specific exercises. **a** Education about good sitting position. **b** The plank position during standing, with both shoulders abducted and externally rotated. **c** Rotary breathing training in squatting. **d**–**f** Active 3-dimensional correction in front of a mirror while the individual is seated

Each subject will undergo outpatient PSSE under the supervision of therapists during 30 min sessions, 5 times per week, for 4 weeks; then, they will undergo another 4-week home-based PSSE regimen implemented under the supervision of their parents, with 30-min sessions performed 5 times a week. The PSSE will include autocorrection in three dimensions, stabilization of the corrected posture, training related to activities of daily living and patient education [35]. The PSSE program will be developed based on the work of Schroth [36, 37] and on the scientific exercise approach to scoliosis [38]. Three-dimensional active correction involves correcting the curvature in the coronal plane, rotation in the horizontal plane, and abnormal physiological curvature in the sagittal plane at the same time. During training, the individuals gradually transition from lying to sitting, and finally to standing. A balance pad or wedge pad will be used for correcting the lateral curvature or hump, and an elastic band will be used to help stabilize the subjects in the corrected posture. Rotational breathing will be involved to correct vertebral rotation as well as asymmetry in the thorax [19]. The subjects will be trained to draw in air to the concave side when inhaling and to expel air first from the convex side when exhaling. At the beginning, the therapists will use their hands to guide the subjects to inhale or exhale correctly. As the subjects become more skilled, breathing under supervision will continue to be required. Meanwhile, the therapists will instruct the subjects on how to integrate their exercises into their daily activities, such as standing and sitting. It will also be necessary to educate the subjects and their parents about the types of scoliosis, possible hazards, and the risk of progression. The PSSE will be guided by two therapists with more than 4 years of experience in the treatment of scoliosis. Some PSSE examples are shown in Fig. 3.

#### Spinal manipulative therapy

In the first 2 weeks, the study group will additionally receive SMT before PSSE 5 times a week. SMT will involve 5 min of evaluation, 10 min of soft tissue relaxation techniques, and 15 min of HVLA manipulation.

Before SMT is performed, it is necessary to evaluate and find the dysfunctional spine segments. Pain and tenderness during palpation or the examination of local tissue are the primary indicators used for identifying the dysfunctional segments [39]. Restricted joint mobility during the spinal examination is also an important indicator [39]. In this study, if the physiotherapist finds any pain, tenderness, or restricted joint mobility when assessing the region from C7 to S1 and the sacroiliac joints, the segments involved will be defined as dysfunctional and recorded as target segments for treatment. For thoracic and lumbar segments, the alignment of the spinous processes is assessed with unilateral or bilateral fingertip contacts. Any potential tenderness or pain around the spinous processes and soft tissues will be recorded. In addition, coupled lateral flexion and rotation movements will be induced on both sides in the extended and flexed positions. For example, the examiner will stand on the subject’s left side, place his right thumb on the right thoracic spinous process, and guide the subject’s upper body with his left hand from a neutral and upright sitting position into rotation and lateral flexion position until the movement reaches the palpating thumb. During the examination, if the subject complains of pain or the examiner observes asymmetric motion between the left and right sides, that means the segments are dysfunctional. For the sacroiliac segments, the subjects will stand barefoot on a firm surface, and the therapist will first palpate the posterior superior iliac spine on both sides and assess the asymmetry as well as the presence of any pain or tenderness during palpation. The positions of the bilateral sacroiliac joint surface projections will be assessed at the same time. The evaluation procedure will be based on that described in chapters 9 to 11 of the palpation techniques book: surface anatomy for physical therapists [40].

After the examination of all segments is completed, the therapist will perform 10 min of soft tissue relaxation on the dysfunctional segments by using rolling manipulation, a form of traditional Chinese massage. The subjects will be instructed by the therapist to lie in the prone position and to relax their mind and body. The therapist will contact the treatment site with the dorsal side of his fifth metacarpophalangeal joints and then sink the shoulders and loosen the wrists, with the elbow joint serving as the support. The forearm will actively swing, driving flexion and extension of the wrist joint and rotation of the forearm. The metacarpophalangeal joints will be rolled back and forth on the treatment site at a frequency of 120–160 times/min [41].

**Fig. 4:**
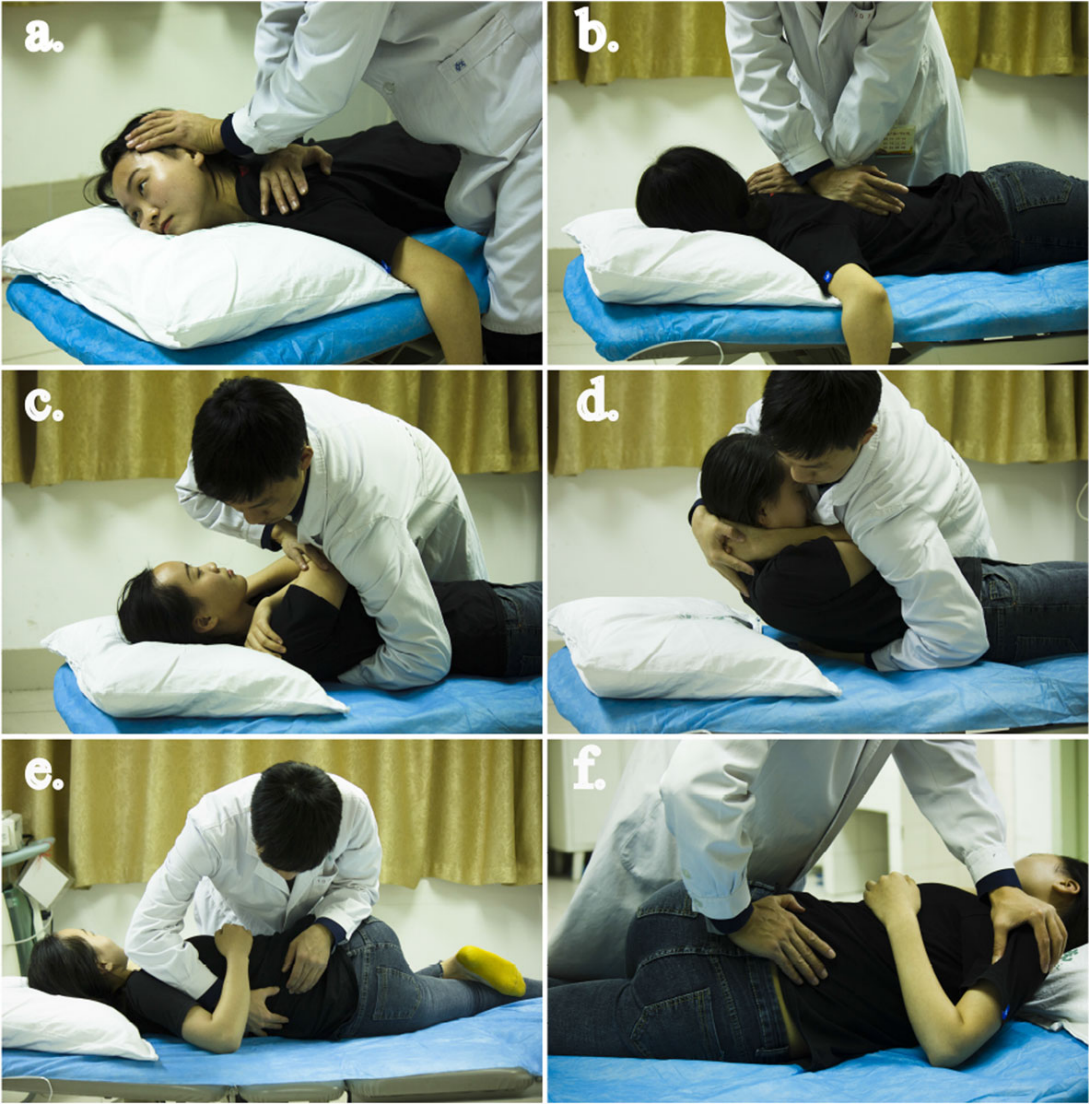
High-velocity, low-amplitude (HVLA) spine manipulation. **a** HVLA for C7-T4 segments while prone. **b** HVLA for T3 to T8 while prone. **c** HVLA for upper thoracic segments while supine. **d** HVLA for lower thoracic segments while supine. **e** HVLA for lumbar segments in lateral lying. **f** HVLA for the sacroiliac joint in lateral lying

After soft tissue relaxation, the therapist will perform HVLA manipulation on the dysfunctional segments to address the subject’s pain, muscle tension, and limited segmental activity. Figure 4 outlines the main HVLA manipulation procedures in our research. For example, Fig. 4c outlines the HVLA technique for adjusting the extension of middle thoracic section. To induce extension with the patient in the supine patient position, the thenar contacts are typically established over the transverse processes of the inferior vertebral segments. The joint to be adjusted is bent into extension over the top of the posterior contact, and the therapist exerts a force posteriorly, using body weight to induce distraction in the joints above the contact. All HVLA manipulation methods will be based on that described in chapter 5 of the chiropractic technique book [42]. The treatment will be stopped immediately if discomfort occurs. One physiotherapist with 10 years of HVLA manipulation experience will perform all the examinations and manipulations.

#### Compliance supervision

To promote subject cooperation, the subjects and their parents will be educated before treatment about the risk of progression of scoliosis, its harmfulness, and the necessity for treatment. We will invite the subjects participating in the study to enter the WeChat group we have established, and a coordinator will manage the WeChat group and regularly share information about scoliosis within the group. In the 4-week period of home-based training, the parents will be required to supervise their children’s training and report the daily training intensity using the WeChat check-in procedure. A physiotherapist will monitor the parents’ reports and provide suggestions and encouragement. When a subject’s compliance begins to falter, their parents will be encouraged through WeChat to encourage the subjects to continue training. If the subjects are able to complete the trial, we will provide free scoliosis consultation services until the subjects are 16 years old.

### Assessment

Age, sex, height, weight, Risser sign, menarche status (for the girls), and type of scoliosis will be recorded at the start of the experiment. The United States Risser staging will be used to quantify skeletal maturity on a scale of 0 to 5 [43]. Stage 0 represents no ossification of the iliac apophysis, whereas stage 5 represents complete ossification and fusion. The scoliosis types will include thoracic, lumbar, thoracolumbar, and S-shaped scoliosis, corresponding to the anatomical site of the spinal deformity [1].

The primary outcome will be the feasibility of performing a full-scale study using these protocols. The time to complete enrollment, recruitment rate, subject retention, and adherence to the treatment allocation will all be important observations. The efficacy of treatment will be the secondary outcome. Spine curvature, rotation of the trunk, and postural symmetry will be assessed by the Cobb angle from X-ray images; the ATR, as measured with a Scoliometer; and 3-DPPs, as measured using a global postural system (GPS), respectively. The SEP will be assessed for evaluating sensorimotor integration, and the SRS-22 will be administered for the quality of life. The Cobb angle will be measured at baseline and at the end of 8 weeks of training. The SEP will be measured at baseline and at the end of 2 weeks of training. The ATR, 3-DPPs, and SRS-22 scores will be measured at baseline and then after 2 weeks, 4 weeks, and 8 weeks of treatment. The mean changes of Cobb angle from baseline to the end will be compared. And the mean difference of other indicators between each two visits will be used for comparative analysis.

### Primary outcomes

The primary outcome of the study will be a judgment about the feasibility of conducting a full-scale RCT. The specific aspects of feasibility that will be monitored are listed below.

#### Integrity of the study protocol

Here, integrity includes the appropriateness of the inclusion criteria, the training of the staff, clinic accessibility for the subjects, the acceptability of the intervention by the subjects and therapists, and the time and facilities required to deliver the interventions. These data will be gathered from interviews with the subjects and therapists.

#### Recruitment and retention

The goal is to enroll at least 80% of the eligible subjects recruited. Once the subjects are enrolled, the goal will be that at least 80% of the subjects attend at least 75% of the treatment sessions and complete at least 75% of the prescribed exercises.

#### Randomization procedure

The appropriateness of the methods used to ensure the blinding of the assessor and the statistical analyst will be determined through post-study interviews in which these individuals will be asked whether they were aware of the group allocation results and whether they felt that the security protocols were reliable.

#### Primary outcome measure

Selection the most appropriate primary outcome measure for a full-scale RCT will be determined using the secondary outcome measures with the largest between-group effect size.

#### Sample size calculation

The sample size needed for a full RCT will be estimated using the effect-size data from this pilot study.

### Secondary outcomes

#### Cobb angle

The Cobb angle will be measured using a standing, full-spine X-ray image in the anteroposterior view. The most tilted vertebrae above and below the curve will be determined first. These vertebrae will be those where the endplates are most tilted towards each other [44]. The angle between the intersecting lines drawn perpendicular to the top of the top vertebra and the bottom of the bottom vertebra will be measured as the Cobb angle.

#### Somatosensory evoked potentials

According to previous studies, the dual peripheral nerve stimulation SEP technique may be a sensitive measure of sensorimotor integration for musculoskeletal disorders [45, 46]. Our research will follow Haavik-Taylor’s protocol [46] for the collection and analysis of SEP in the upper limbs, especially regarding the parameters of the dual stimulation of the median and ulnar nerves.

All subjects will undergo SEP assessment twice at baseline and at least 6 h after the last treatment session after 2 weeks of training. In each assessment, three SEP trials (average 1000 sweeps) will be carried out in a random order, including the stimulation of the median nerve individually (M), stimulation of the ulnar nerve individually (U), and simultaneous stimulation of both nerves (MU). The stimuli consist of electrical square pulses that are 1 ms in duration and delivered at a rate of 2.47 Hz, at the motor threshold. All the stimulating electrodes, recording electrodes and their reference electrodes will be placed according to the recommendations of the International Federation of Clinical Neurophysiologists (IFCN) [47]. During the recordings, the lights in the room will be dimmed, and the subjects will be asked to lie supine on the bed with their limbs relaxed and eyes closed.

According to the recommendations of IFCN, the SEP amplitudes will be measured from the peak of interest to the preceding or succeeding trough. The SEP latencies will be measured at the peak of the waveform of interest. The amplitudes and latencies of peripheral N9, spinal N11 and N13, far-field P14 and N18 potentials, parietal N20 (N20–P25 complex), and frontal N30 (P22–N30 complex) will be identified and analyzed. In addition, the MU/M + U SEP peak ratios will be calculated. An assessor with 4 years of experience using the EMG equipment will complete all the subjects’ assessments.

#### Angle of trunk rotation

A Scoliometer (Fabrication Enterprises Inc., New York, USA) will be used to quantify the degree of rotation of the trunk. The subjects will be instructed to slowly bend forward to approximately 90° with their feet together, knees straight, arms dangling, and hands together [48]. The Scoliometer will be centered on the spinous process and placed vertically in the most obviously deformed part of the subject’s back. The therapist will ensure that the subject’s back is horizontal and then record the angle of trunk rotation.

#### Three-dimensional postural parameters

A GPS (Chinesport, Italy) will be used to assess the 3-DPPs. The device has a unit for podoscopic analysis, a unit for postural analysis and a stability measuring platform, and it comes with an image acquisition system and custom software. The camera of the image acquisition system will be positioned 107 cm from the ground and 190 cm from the subject. The protocol published by Youssef’s group [49] will be used to analyze each subject’s posture in the coronal and sagittal planes.

##### Assessment environment

The 3-DPPs’ assessments will take place in a private, warm, well-lit room with flat-white walls.

##### Preparation of the GPS

The GPS device must be adjusted to a standard position before the assessment.

##### Preparation of the subjects

The subjects will be instructed to remove their clothes and wear only their underwear. They will then stand naturally with their feet shoulder-width apart while 11 round, reflective, red stickers 5 mm in diameter are attached to their body.

##### Marker placement

1. The posterior view markers will include eight round, reflective, red stickers and will be placed on the following anatomical landmarks: right and left acromia, two spinous processes (C7, S1), the right and left inferior angles of the scapula, and the posterior superior iliac spine.
2. The right lateral view markers will include three round, reflective, red stickers and will be placed on the following anatomical landmarks: right tragus, right canthus, and right anterior superior iliac spine.

##### Photo acquisition

The subjects will be asked to place their feet on standard footprints, to stand naturally and to take 3 deep breaths to relax the body. The subjects’ back and right side will then be photographed.

##### Posture parameter analysis

The images will be processed by the GPS system’s software to measure the angles and distances between the markers. Estimates of the sagittal head angle, craniovertebral angle, protracted shoulder angle, shoulder alignment, scapula asymmetry, right and left waist angle, frontal pelvic tilt, sagittal pelvic tilt right, C7 alignment, and S1 alignment will be generated. Fortin’s method [50] will be used to calculate the angles and distances*.* Some details are shown in Additional file 3.

The same assessor with 4 years of experience using the GPS equipment will complete all of the subject assessments. The reliability and validity of this device has been verified in previous studies [51, 52].

#### SRS-22

The SRS-22 questionnaire is widely used for assessing the quality of life of individuals with scoliosis. It has five dimensions: functional activity, pain, self-image, psychological status, and satisfaction with treatment. The first four dimensions each contain five items, and the treatment satisfaction dimension contains two items. Each item has a maximum score of 5 and a minimum score of 1 point. The score for a dimension is the average of the scores for the items included in the dimension, so the scores range from 1 to 5. We will calculate the average score for each dimension and the total score for all dimensions. We will use the simplified Chinese version [53] of the questionnaire with question 15 modified to be filled in by the parents [54].

### Adverse events

Adverse events during SMT can be classified as mild (transient effects lasting fewer than 24 h), moderate (requiring medical treatment), or severe (requiring hospitalization) [55]. Severe adverse events are rarely reported with SMT [56, 57], but mild and moderate adverse events are common, though self-limiting. Most occur within 24 h and resolve without treatment within 72 h [56]. Almost all of the severe adverse events attributable to SMT are related to cervical manipulation and underlying pre-existing pathologies [57, 58].

In this trial only SMT of the thoracic, lumbar, and sacral segments will be administered. The cervical segments will not be involved. And subjects with pre-existing pathologies detected in systematic physical examinations and spinal X-ray examinations will be excluded. Considering that all the subjects in this study will be adolescents, the HVLA process will be performed carefully and the force of the manipulation will be adjusted according to the age and condition of the subject. Adverse events should therefore be few and relatively minor. In the first 2 weeks, the therapist will inquire about adverse events daily via telephone. In the next 6 weeks, weekly follow-up via telephone will be conducted by the same therapist. Subjects will also be required to contact the therapist if they experience pain, transient headache, or any other discomfort that may be relevant to treatment. If the discomfort does not disappear within 24 h after treatment, the SMT will be discontinued. Any adverse events observed will be recorded and reported to the supervising ethics committee, regardless of their severity. Additionally, both predictable potential adverse events (e.g., crying, pain, or transient headache, etc.) and unpredictable adverse events hindering the health of subjects happened in this trial will be reported truthfully in future publications.

### Sample size

G*Power 3.1.9.7 was used to execute the power calculations. The change of the Cobb angle was chosen as the primary outcome indicator. Referring to the two studies from Zheng (*d* = 0.8) [59] and Liang et al. (*d* = 1.2) [60], we set the value of effect size *d* = 1.0 in our study. With a significance level of 5%, 17 subjects are needed in each group to reach a power of 80%. Allowing for a dropout rate of 15%, a total of 40 subjects will be recruited with 20 subjects for each.

### Data collection, data management, and statistical analysis

The primary outcome measures will be tabulated and interpreted with respect to the goals established a priori.

To estimate the sample size required for a future full-scale RCT, between-group effect sizes and their 95% confidence intervals will be calculated by applying Hedges’ correction. A minimum power of 80% will be used to detect changes in the Cobb angles, ATRs, 3-DPPs and SRS-22 scores (*α* = 0.05). The sample size will be increased to allow for an estimated 20% dropout rate. Statistical methods for the secondary outcome measures will be evaluated via comparisons of changes between groups.

The data will be double entered into EpiData 3.1 software (The EpiData Association, Odense, Denmark) and then transferred to version 22.0 of the SPSS software suite (IBM, Armonk, NY, USA) for the analyses. The threshold for significance will be taken as *α* ≤ 0.05. A statistician who will be blinded to the allocation results will analyze all the data. The continuous variables will be presented as means ± SDs, and the categorical variables will be presented as ratios. The baseline readings will be compared using independent samples *t* tests or chi-squared tests. For the Cobb angles and SRS-22 scores, independent samples *t* tests will be used to compare the two groups, and paired *t* tests will be used for within-group comparisons. For the M, U, MU, and M+U SEP peak amplitudes and latencies, nonparametric Wilcoxon signed-ranks tests will be used to compare these values between and within the groups. For the 3-DPPs and ATR, repeated measured analysis of variance will be used for the between-groups and within-groups comparison. Furthermore, we will perform subgroup analysis by age group (age < 13 years and age ≥ 13 years) [2, 33]. To deal with the missing data, a sensitivity analysis and weighted estimating equations will be used as the primary approach [61].

### Monitoring and auditing

A monitoring board, including a physician, two physiotherapists, two assessors, and a statistician, will be responsible for reviewing all the data and will be allowed to conduct an audit of the trial at any time. The monitoring board will be independent from the sponsor and have no conflicts of interest. Besides the monitoring board, the Independent Supervisory Committee (ISC) in our institution will undertake the duties of monitoring the progress of experiments and data quality. It consists of one independent chair and at least six members including clinical experts, statisticians, patient, and public involvement representatives. All the data will be securely stored on EpiData software.

### Interim analysis

An interim analysis will be conducted by statisticians when 20 subjects complete the experiment. In our study, the primary endpoint will be analyzed via the change of Cobb angle. In order to maintain the cumulative type I error rate at the planned 0.05, the Lan-Demets spending function method [62] with the O’Brien-Fleming spending function [63] will be used. The preplanned *P* value for stopping the trial owing to efficacy will be 0.00305. Our research team will discuss the results of the interim analysis with the ISC and the monitoring board to determine whether to stop the trial early. Besides, when a serious adverse event occurs in our study, the trial will also be terminated early.

### Confidentiality

A nonidentifiable unique trial number for all subjects will be used to maintain confidentiality for all data. Only the designated trial investigators will have the authority to access the final data.

### Access to data

Only the main investigators will have the authority to access the final trial dataset. Anonymized data will be delegated to statistician during and after the trial.

### Ancillary and posttrial care

After the trial is completed, we will continue to provide follow-ups for all subjects and provide necessary physical therapy recommendations if needed.

### Dissemination policy

The final results of the study will be published in a scientific journal and will be disseminated at a relevant international conference. We will report our study following the CONSORT statement guidelines updated in 2010. The primary members of the research team will be named coauthors on all related publications. We do not intend to use any external professional writers for publication.

## Discussion

Although AIS has been diagnosed and treated for many years, nonoperative treatment approaches have not been thoroughly investigated [2]. For patients who are skeletally immature, the “watch and wait” approach followed by bracing (when the Cobb angle exceeds 25°) has become the standard treatment in the USA [64], though some Europeans have advocated for more proactive physical therapy interventions to maintain or even reduce curvatures [65, 66]. Due to the lack of scientific evidence, the recommended treatment for mild AIS is only PSSE, but the effect is not always satisfactory [1].

SMT is a popular and effective treatment for nonspecific neck or back pain [20, 21]. Symptoms such as pain and limited mobility can often disappear quickly after SMT, typically after a few weeks of treatment [67]. It is commonly used for scoliosis, but its efficacy remains unclear.

Thus, we designed this pilot RCT to include 2 weeks of SMT and 8 weeks of PSSE. The hope is that the combination can help reduce the Cobb angle, improve trunk symmetry, and improve quality of life to a greater extent than can PSSE alone. Our expectations are not unreasonable because pain and restricted joint mobility in the spine are the common manifestations of spinal dysfunction, including scoliosis. SMT has been shown to be effective in relieving pain along the spine through inhibition mechanisms mediated by the central nervous system [68]. Pain relief reduce movement limitations and helps patients achieve better motor performance. SMT can help improve spine mobility, segmental range of motion, dysfunction of the facet joints, and muscle imbalances [22]. Improved bone alignment and more symmetrical muscle states will lead to a better structural foundation for PSSE. Indeed, there is evidence that SMT can also improve motor performance by improving sensory-motor integration [27–32]. PSSE training after SMT is likely to yield better motor control and improve the efficiency of the exercise [32].

### Limitations of this study

It is important to note, however, that this pilot RCT will not be double-blinded. Only the frequency and not the quality of the home exercise will be recorded. The follow-up period may also be insufficient, as it is only 4 weeks, and there will be no longer-term observations.

## Trial status

Ongoing recruitment. This is protocol version 1.0; the protocol version date is October 30, 2018. Subject recruitment began on November 01, 2019, and will end on November 01, 2021.

## Supplementary Information

**Additional file 1.** SPIRIT 2013 Checklist: Recommended items to address in a clinical trial protocol and related documents.

**Additional file 2.** The items from the World Health Organization Trial Registration Data Set.

**Additional file 3.** Posture parameters of GPS and angle and distance calculation method.

## Data Availability

Data and materials will not be made public until they are published in a peer-reviewed international journal. After publication, the final trial dataset will be available from the principal investigator upon reasonable request by email.

## Abbreviations

AIS: Adolescent idiopathic scoliosis
ATR: Angle of trunk rotation
CONSORT: Consolidated standards of reporting trials
GPS: Global posture system
HVLA: High-velocity, low-amplitude
IFCN: International Federation of Clinical Neurophysiologists
ISC: Independent Supervisory Committee
PSSE: Physiotherapeutic scoliosis-specific exercises
RCT: Randomized controlled trial
SMT: Spinal manipulative therapy
SEP: Somatosensory evoked potentials
SOSORT: International Scientific Society on Scoliosis Orthopedic and Rehabilitation Treatment
SPIRIT: Standard Protocol Items Recommendations for Interventional Trials
SRS-22: The 22-item Scoliosis Research Society Outcomes Questionnaire
3-DPPs: Three-dimensional postural parameters
