# Supplementary material for: Physiotherapeutic scoliosis-specific exercises performed immediately after spinal manipulative therapy for the treatment of mild adolescent idiopathic scoliosis: study protocol for a randomized controlled pilot trial
Source: Trials. 2021 Jan 14;22:58. doi: 10.1186/s13063-020-05000-y (PMC7807706; doi:10.1186/s13063-020-05000-y)
Supplement: Supplementary file 2 — Additional file 2. The items from the World Health Organization Trial Registration Data Set. [file 13063_2020_5000_MOESM2_ESM.docx]

**The items from the World Health Organization Trial Registration Data Set**

| Data category | Information |
| --- | --- |
| Primary registry and trial identifying number | ChiCTR1900027037 |
| Date of registration in primary registry | October 29, 2019 |
| Secondary identifying numbers | Not applicable |
| Sources of monetary or material support | The Tongji Hospital Foundation supports this work (grant number 2201300754). |
| Primary sponsor | Tongji Hospital, Tongji Medical College, Huazhong University of Science and Technology |
| Secondary sponsor(s) | Not applicable |
| Contact for public queries | Xiaolin Huang ([xiaolinh2006@126.com](mailto:xiaolinh2006@126.com)); Nan Xia ([xianan@tjh.tjmu.edu.cn](mailto:xianan@tjh.tjmu.edu.cn)); |
| Contact for scientific queries | Xiaolin Huang ([xiaolinh2006@126.com](mailto:xiaolinh2006@126.com)); Nan Xia ([xianan@tjh.tjmu.edu.cn](mailto:xianan@tjh.tjmu.edu.cn)); |
| Public title | Physiotherapeutic Scoliosis-Specific Exercises immediately after Spinal Manipulation for the treatment of mild adolescent idiopathic scoliosis: Study Protocol for a randomized controlled pilot trial |
| Scientific title | Physiotherapeutic Scoliosis-Specific Exercises immediately after Spinal Manipulation for the treatment of mild adolescent idiopathic scoliosis: Study Protocol for a randomized controlled pilot trial |
| Countries of recruitment | China |
| Health condition(s) or problem(s) studied | Spinal disease |
| Intervention(s) | 2-week spinal manipulation plus 8-week Physiotherapeutic Scoliosis-Specific Exercises vs. 8-week Physiotherapeutic Scoliosis-Specific Exercises |
| Key inclusion and exclusion criteria | Inclusion criteria: (1) Age 10 – 18 years; (2) Diagnosed with idiopathic scoliosis; (3) Cobb angle 10 – 25°; (4) Risser sign 0 – 2. (5) Agree not to receive any other treatments for scoliosis such as brace and insoles during the study period.  Exclusion criteria: (1) Contraindications for exercise such as cardiovascular or respiratory insufficiency; (2) Contraindications for Spinal manipulation: inflammation, infection, advanced degeneration, congenital malformations, trauma, cerebrovascular anomalies; (3) A history of previous spinal surgery; (4) A history of previous scoliosis treatment involving surgery, a brace, exercise, etc. |
| Study type | Interventional. Allocation: randomized; Masking: no blinding; Assignment: parallel. Primary purpose: treatment |
| Date of first enrolment | November 29, 2019 |
| Target sample size | 40 |
| Recruitment status | Ongoing recruitment |
| Primary outcome(s) | The feasibility of conducting a full-scale randomized clinical trial: Integrity of the Study Protocol, Recruitment and Retention, Randomization Procedure, Primary Outcome Measure. |
| Key secondary outcome | (1) Cobb angle; (2) Somatosensory evoked potentials; (3) Angle of trunk  rotation; (4) Three-dimensional posture parameters; (5) Scoliosis Research Society Patient Questionnaire-22. |
