# Supplementary material for: Physiotherapeutic scoliosis-specific exercises performed immediately after spinal manipulative therapy for the treatment of mild adolescent idiopathic scoliosis: study protocol for a randomized controlled pilot trial
Source: Trials. 2021 Jan 14;22:58. doi: 10.1186/s13063-020-05000-y (PMC7807706; doi:10.1186/s13063-020-05000-y)
Supplement: Supplementary file 3 — Additional file 3. Posture parameters of GPS and angle and distance calculation method. [file 13063_2020_5000_MOESM3_ESM.docx]

**Posture parameters of GPS and angle and distance calculation methods**

| **Body segment** | **Posture parameters** | **Angle or distance calculation** |
| --- | --- | --- |
| **Head and neck** | 1. Sagittal head angle | The angle formed by a line connecting the canthus of the eye with the tragus of the ear, and a horizontal line |
|  | 2. Craniovetebral angle | The angle formed by a line connecting the tragus of the ear with C7, and a horizontal line through C7 |
| **Shoulders and scapula** | 3. Protracted shoulder angle | The angle formed by a line connecting C7 with the [acromion](javascript:;), and a horizontal line through C7 |
|  | 4. Shoulder alignment | The angle with the horizontal of a line connecting the left and right [acromion](javascript:;) markers |
|  | 5. Scapula asymmetry | The angle with the horizontal of a line connecting the left and right inferior angles of the scapula |
| **Lumbar** | 6. Waist angle Right | The angle formed by lines drawn through the upper end of the right waist to the center of the right waist, and the center of the right waist through the lower end of the right waist. |
|  | 7. Waist angle Left | The angle formed by lines drawn through the upper end of the left waist to the center of the left waist, and the center of the left waist through the lower end of the left waist. |
| **Pelvic** | 8. Frontal pelvic tilt | The angle with the horizontal of formed by a line joining the two posterior superior iliac spines |
|  | 9. Sagittal pelvic tilt R | The angle formed by the horizontal and by the line joining the right posterior superior iliac spine and anterior superior iliac spine. |
| **Trunk** | 10. C7 alignment | Distance between the midline and the vertical line passing through C7 |
|  | 11. S1 alignment | Distance between the midline and the vertical line passing through S1 |
